# Silicone Nanofilament Support Layers in an Open-Channel System for the Fast Reduction of *Para*-Nitrophenol

**DOI:** 10.3390/nano11071663

**Published:** 2021-06-24

**Authors:** Noah U. Naef, Stefan Seeger

**Affiliations:** Department of Chemistry, University of Zurich, 8057 Zurich, Switzerland; noah.naef@chem.uzh.ch

**Keywords:** nanomaterials, open channel, catalysis

## Abstract

Chemical vapor phase deposition was used to create hydrophobic nanostructured surfaces on glass slides. Subsequently, hydrophilic channels were created by sputtering a metal catalyst on the channels while masking the outside. The surface tension gradient between the hydrophilic surface in the channels and the outside hydrophobicity formed the open-channel system. The reduction of *para-*nitrophenol (PNP) was studied on these devices. When compared to nanostructure-free reference systems, the created nanostructures, namely, silicone nanofilaments (SNFs) and nano-bagels, had superior catalytic performance (73% and 66% conversion to 55% at 0.5 µL/s flow rate using 20 nm platinum) and wall integrity; therefore, they could be readily used multiple times. The created nanostructures were stable under the reaction conditions, as observed with scanning electron microscopy. Transition electron microscopy studies of platinum-modified SNFs revealed that the catalyst is present as nanoparticles ranging up to 13 nm in size. By changing the target in the sputter coating unit, molybdenum, gold, nickel and copper were evaluated for their catalytic efficiency. The relative order was platinum < gold = molybdenum < nickel < copper. The decomposition of sodium borohydride (NaBH_4_) by platinum as a concurrent reaction to the *para*-nitrophenol reduction terminates the reaction before completion, despite a large excess of reducing agent. Gold had the same catalytic rate as molybdenum, while nickel was two times and copper about four times faster than gold. In all cases, there was a clear improvement in catalysis of silicone nanofilaments compared to a flat reference system.

## 1. Introduction

Microfluidic devices are widely used in the biomedical field for applications such as nucleic acid and protein analysis, cellular studies, binding affinity measurements, or immunoassays [1,2]. However, only a few researchers have focused on the advantages of heterogeneous catalysis in microfluidic devices [3]. Depositing a heterogeneous catalyst on the walls of microfluidic devices results in an exceptional catalyst-to-reagent ratio [3]. In addition, microfluidic devices allow for the tight control of the reaction time via the flow rate and size of the system [3]. Shorter reaction times result in reduced time for side reactions [3]. Removing the product from the system and collecting it under appropriate conditions prevents undesired further reactions or decomposition that could occur if the product had longer exposure to the reaction conditions. This has led, in some cases, to microfluidic systems that outperformed batch reactions in terms of yield and selectivity [4].

Open-channel systems are defined as systems with at least one liquid/air or liquid/liquid interface, with the former being the most common case [5]. Borders and channels are created by manipulating the surface energy and/or morphology, facilitating the flow inside the channels with capillary effects and hydrophilic surfaces and discouraging the wetting of the outside with a hydrophobic surrounding [6,7]. The construction of open-channel systems does not require the steps of closed-channel microfluidic device manufacturing [6]. Silicone nanofilaments (SNFs) are an ideal tool for producing open-channel devices. SNFs are easily created on surfaces by the droplet-assisted growth and shaping (DAGS) synthesis approach [8]. They are superhydrophobic, have good chemical stability and high roughness and can be created as a transparent coating [9]. Their superhydrophobicity provides solid borders for the open chip over an extended time due to their chemical stability. Surface roughness, as defined by Wenzel, is the amplification of the underlying surface hydrophobicity [10,11]. The high roughness inside the channels provides an amplified catalytical surface once the heterogeneous catalyst is installed [12]. This is significant, as the high catalyst-to-reagent ratio is a main motivation for performing reactions in microdevices.

The reduction of *para-*nitrophenol (PNP) to *para-*aminophenol (PAP) is of commercial interest, because PAP has uses in photography and as a building block in pharmaceuticals such as paracetamol [13]. Besides its practical implications, the reaction has gained popularity as model reaction for catalyst development due to its clean reaction pathway, which is readily observable with UV spectroscopy [14]. It proceeds quickly at room temperature and has been shown not to occur in the absence of a metal catalyst [15]. The reaction starts by transferring hydrides form a reducing agent, typically NaBH_4_, on to the metal catalyst to form a surface hydrogen species [16]. When PNP adsorbs onto the catalyst, a three-step reduction occurs and PAP is released as product [17]. The most abundantly featured metal for this reaction is gold, generally in the form of stabilized nanoparticles, as described in Zhao et al. [14]. Solid supports with many different stabilizing materials have been used, including organic ligands [18], polymers [19], peptides [15] and metal oxides [20]. The list of metals with activity in this reaction includes gold [14,15,16,17,18,19,20], copper [21], silver [22,23], palladium [17,24], nickel [25,26], molybdenum oxide [27] and iron oxides [28,29].

In this study, hydrophobic surfaces, SNFs and bagel nanostructures were created. The advantage of nano-roughness for the catalytic rate was studied. The stability of the created nanostructures under the applied reaction conditions was confirmed by scanning electron microscopy (SEM). Transition electron microscopy (TEM) of the detached SNFs was used to study how the metal catalyst was dispersed on the SNFs. Catalytic studies were conducted with molybdenum, gold, nickel and copper on SNFs and a nanostructure-free reference surface.

## 2. Materials and Methods

### 2.1. Materials

Vinyltrichlorosilan (VTCS) was obtained from abcr GmbH (Karlsruhe, Germany) and stored under nitrogen.Coverslips of size 26 × 76 mm were purchased from Menzel (Braunschweig, Germany) and *para*-aminophenol, *para*-nitrophenol and sodium borohydride were obtained from Sigma-Aldrich (St.Louis, MO, USA) and used as received. The holes in the glass slides were drilled using a KL450 ultrasonic drill (AQUARUS, Pappenheim, Germany). Sputtering targets (Mo, Pt, Ni, Cu, Au) were obtained from BALTIC Preparation e.K (Wetter, Germany). Sputtering was conducted with a CCU-010 HV coating unit (Safematic, Zizers, Switzerland). BLAUBRAND^®^ intraMARK (Brandt GMBH, Wertheim, Germany) 5 µL capillaries were purchased from the University of Zurich supply shop. An IPC high-precision multichannel dispenser (ISM930C) was used as peristaltic pump (Ismatec, Wertheim, Germany). Double distilled water was from a glass double distillery apparatus (GFL, Burgwedel, Germany) and was used to treat the slides prior to coating (SNFs, bagels); all other solutions were prepared from ultrapure water (Simplicity^®^ UV system) (Millipore, Billerica, MA, USA). SEM images were taken on a 450 Zeiss Gemini (Zeiss, Jena, Germany). TEM was performed using a FEI Tecnai G2 Spirit (FEI company, Hillsboro, OR, USA).

### 2.2. Methods

#### 2.2.1. Drilling

Inlet or outlet holes of 3 mm diameter were drilled into the Menzel glass slides, prior to any coating, at the appropriate position.

#### 2.2.2. Surface Silanization

Six Menzel coverslips were activated twice for 4 min in oxygen plasma (10 W, 10 Scc). The slides were placed in a custom-made gas phase reaction chamber (volume of 6.5 L) and the chamber was flushed for 1 h with dry nitrogen. The flow was stopped and 600 µL of VTCS were injected through a septum onto a silane holder placed in the middle of the reaction chamber. After 1 h, the slides were removed from the reaction chamber.

#### 2.2.3. SNF Coating

Six Menzel coverslips were sonicated for 30 min in 1 M of NaOH (1 L) at 50 °C with 10 W. They were then washed twice for 10 min in double distilled water (1 L) at 50 °C with 10 W sonication. The activated slides were dried with dry nitrogen gas and placed in a custom-made gas phase reaction chamber (volume of 6.5 L). The reaction chamber was flushed for 2 h at 40% relative humidity with nitrogen gas. The flow was stopped and 600 µL of VTCS were injected through a septum onto a silane holder placed in the middle of the reaction chamber. The reaction was left overnight.

#### 2.2.4. Silicone Bagel Coating

Six Menzel coverslips were sonicated for 30 min in 1 M of NaOH (1 L) at 50 °C with 10 W. They were then washed twice for 10 min in double distilled water (1 L) at 50 °C with 10 W sonication. The activated slides were dried with dry nitrogen gas and placed in a custom-made gas phase reaction chamber (volume of 6.5 L). The reaction chamber was flushed for 2 h at 60% relative humidity with nitrogen gas. The flow was stopped and 270 µL of VTCS were injected through a septum onto a silane holder placed in the middle of the reaction chamber. The reaction was left for 1 h.

#### 2.2.5. Plasma Treatment

For flow devices, a cellophane negative was placed on top of the coated slide and attached by heating the slide to 40 °C for 2 min. Slides intended for batch measurements were used directly. The slide was then placed in a plasma reaction chamber. The chamber was flushed with oxygen gas for 4 min before the plasma was started (40 s, 10 W, 10 Scc). The success of the oxidation was tested by placing a water droplet on the exposed area.

#### 2.2.6. Sputtering

For flow devices the negative was placed on top of the coated coverslip and all were placed inside a sputter coater. The targets of less noble metals, such as copper, molybdenum and nickel, were scrubbed with iron wool just prior to use. The surface was sputtered to 20 nm thickness. A second mirrored sample with a 3 mm hole at the other end was produced in the same fashion.

#### 2.2.7. Contact Angle Measurement

The static contact angle was measured with the contact angle system (OCA) and software provided by Dataphysics (SAC202 Version 2.3.9 build 46 with Image processing unit version 2.03 Build 5, dpi_Gnl.dll version 1.0.6 build 7, dpi_Sys version 1.06 build 7 dpi Editor.dll version 1.0.6 build 7, dpi_Interp.dll version 1.0.3 build 3 dps_s version 1.0.0 build 1, Dataphysics Filderstadt Germany). The droplet volume was 10 µL. The contact angle was measured at a minimum of 4 positions on both sides of two slides.

#### 2.2.8. Open-Channel Setup

The mirrored coverslips were positioned 0.15 mm apart by two cellophane strips along the sides and held stationary with two paperclips. The exit was placed on top of a capillary punched through a septum into a round-bottom flask. A second capillary, connected to a tube further connected to a peristaltic pump, was punched through the septum to provide suction.

#### 2.2.9. Catalysis on the Open-Channel System

Distilled water was added to PNP (9–14 mg) to create a 20 mM stock solution. The dissolving process was accelerated by immersing the solution in a 50 °C hot water bath and applying sonication at 10 W. The solution cooled to room temperature, then 6.5 mg of NaBH_4_ were weighed out in a 10 mL glass vial. Next, 2.950 mL of distilled water were added, followed by 450 µL 20 mM of stock solution. The solution was quickly shaken and then run through the SNF open-channel system by continual addition of 40 µL droplets to the inlet. By stopping the time and weighing the collected reaction solution in the collecting flask, the flow rate was calculated. Between cycles, the surface was rinsed with distilled water until no yellow coloring could be observed and then dried.

#### 2.2.10. Analysis of PNP Reduction

The reaction solution (9 µL) was diluted in a 1 mL 250 mM potassium hydroxide solution. Conversion was determined as the decrease of absorption at 405 nm compared to a PNP standard curve.

#### 2.2.11. Scattering Electron Microscopy (SEM)

Samples for SEM were sputtered with 10 nm platinum using a CCU-010 HV coating unit. SEM analysis was performed on a Zeiss Supra 450 VP at 3 kV using the SE2 detector (Jena, Germany).

#### 2.2.12. Transmission Electron Microscopy

Five milligrams of coated glass were submerged in 0.2 mL of ethanol and sonicated at 40 °C at 10 W strength for 10 min. Then, 10 µL of the suspension was drop cast on a copper TEM grid. The grid was dried with nitrogen gas and the procedure repeated six times. The size of the catalyst particles and SNFs was calculated by measuring 15 randomly selected particles and 3 diameters and comparing them to the size bar using Fiji.

#### 2.2.13. Batch Setup

A catalyst piece of surface area corresponding to the chip setup was cut off and placed in a custom-made holder (80 × 30 × 3 mm). Then, 1.7 mL of reaction solution containing 50 mM of NaBH_4_ and 2.65 mM of PNP were added. The reaction was performed at room temperature without additional cooling. The reaction solution was homogenized ~10 s prior to taking a sample by taking it up in a 2 mL syringe and redispersing it into the reactor. The 9 µL samples were taken and diluted in a 1 mL 250 mM KOH solution for analysis.

## 3. Results and Discussion

### 3.1. Setup of the SNF Open-Channel System

**Figure 1:**
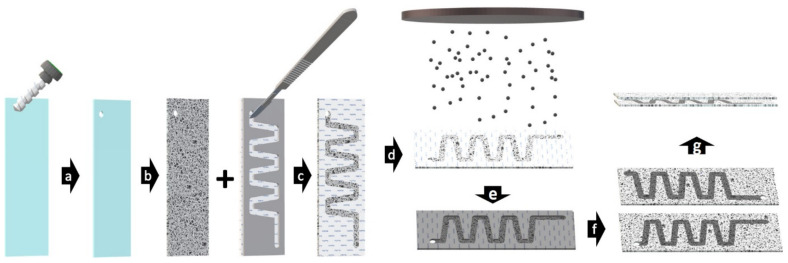
Production steps of the open-channel device: drilling of the inlet/outlet hole (**a**), surface modification (**b**), cutting of the mask (**c**), optional plasma step (**d**), sputtering of the metal catalyst (**e**), removal of the mask and repetition of the process (**f**) and assembly of the device (**g**).

The open-channel system was created as shown in Figure 1. A 3 mm diameter hole was created with an ultrasonic drill, as doing so later would damage the coating (a). The slide was coated to obtain the desired surface morphology (b). A mask was cut from cellophane using a template to improve the consistency and placed on the coated slide (c). The mask was firmly attached by heating slide and mask for 2 min at 40 °C (c). If a SiOH surface chemistry was desired, the masked slide would be placed in the oxygen plasma chamber prior to sputtering (d). After sputtering, the surface was covered with a 20 nm layer of metal catalyst (e). The mask was removed and the process repeated to obtain two mirrored slides (f) that could be arranged on top of one other to provide a catalytic surface and hydrophobic interactions that direct the flow of the reaction solution from top and bottom (g).

**Figure 2:**
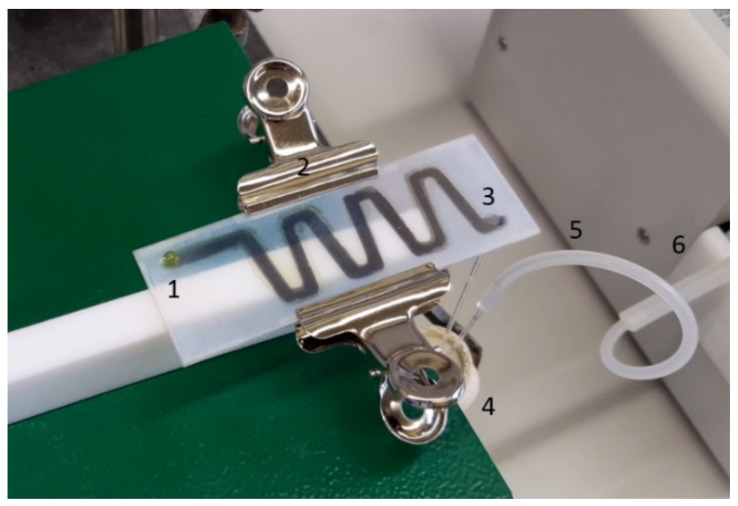
Setup of the SNF open-channel system with an inlet hole on top (**1**). Both slides are held in position by two clamps (**2**). An outlet on the bottom (**3**) and a capillary were installed to suction the product containing solution into the collection flask (**4**). The suction is provided by a second capillary connected with tubing (**5**) to a peristaltic pump (**6**).

Two coated glass slides with mirrored metal sputtered channels of 3 mm diameter were fixed on top of each other with clamps. Two cellophane strips were placed between the glass slides, where they were held in position by the clamps to create a ~0.15 mm gap (Figure 2). This gap was found to be vital to enable the reaction solution to go through the chip. The entry was positioned on top and the exit on the bottom end. A capillary was installed to bring the reaction solution through a septum into a collecting flask. A secondary capillary was installed in the septum and connected to a pneumatic pump to create suction inside the collecting flask.

A volume of 73 µL was required to wet the entire path channel. The channel length was 16.3 cm, resulting in 9.0 cm^2^ of surface area when the top and bottom were combined. Using the surface area of the device, its volume and the PNP concentration (2.65 mM), a catalytic surface to reagent ratio of 4.65 cm^2^/µmol could be calculated (see Supplementary Materials, Equation S1). The ratios were higher for SNF and bagel devices, because their nanoscale roughness increased the catalytic surface.

### 3.2. Different Coatings

To establish the effect of surface morphology on catalysis, three surfaces of different nano-roughness were created by changing the activation of the glass slides and the parameters of the chemical vapor deposition procedure. All surface types were characterized with SEM and contact angle measurements to evaluate their roughness and actual morphology. The conversion over five runs amounted to a total of at least 1.1 mL of solution run through the setup. The hydrophobic surface was tested on multiple replicas, because the weak hydrophobicity on the silanized surface would dissipate after one or two runs.

**Figure 3:**
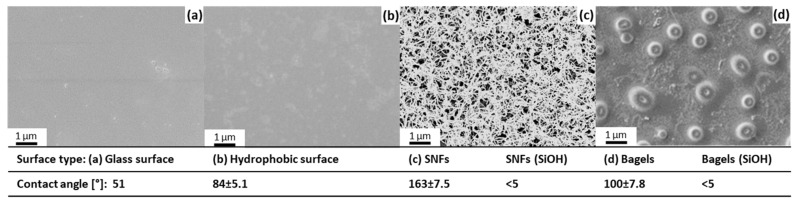
Characteristics and performance of different morphologies and surface chemistries (vinyl or SiOH).

A hydrophobic surface without nanostructures but with surface silanization, as indicated by the increased contact angle of 84 ± 5.1° compared to the 51° original glass surface, was used as reference (Figure 3). The successful creation of SNFs and bagels was confirmed by SEM, while the contact angle provides quality control without having to break down the sample for SEM microscopy. The effects of these surface morphologies in the context of the conversion and flow rate are discussed based on the individual runs.

**Figure 4:**
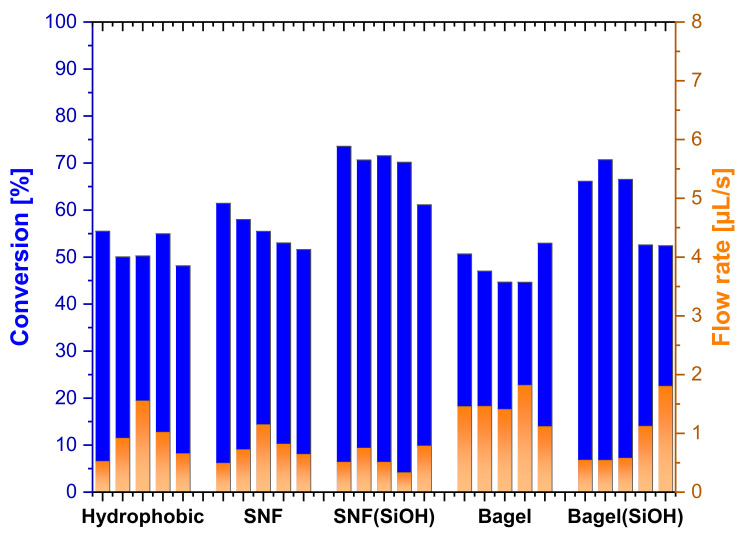
Conversion and flow rate of the individual runs for the hydrophobic control, as well as SNF and bagels with (SiOH), and without plasma treatment prior to sputtering 20 nm of platinum.

SNF coatings showed a higher conversion at comparable flow rates than the hydrophobic surface used as a reference (60 ± 2% at 0.49 µL/s to 52 ± 3% at 0.52 µL/s) (Figure 4). Leakage on the hydrophobic slides was particularly problematic in the case of platinum, because more than any other metal tested later, platinum visibly shows strong gaseous hydrogen evolution upon contact with the catalyst. The formation of hydrogen from sodium borohydride catalyzed by platinum creates a local pressure, particularly where the reaction solution is added, forcing the solution to cross the channel borders. A notable increase in conversion was observed with oxidized SNFs (SNF(SiOH) 74% at 0.52 µL/s to 52 ± 3% at 0.52 µL/s) (Figure 4). This can easily be explained by the improved penetration of the catalyst layer. While SNFs are hydrophilic on the surface due to the metal catalyst sputtered on top of them, they might not be wetted throughout the entire layer as the catalyst is sputtered from the top. In the case of hydrophobic SNFs with platinum, the lower part might be blocked with air or potentially hydrogen gas and the reaction solution essentially floats over the top, providing lower conversions than in the case of completely wetted SNFs.

Applying silicone structures with a bagel shape lead to weaker surface amplification. However, with a contact angle of 100 ± 7.8° the hydrophobic properties were sufficiently strong to last over five runs (Figure 4). The conversion difference between SiOH and vinyl was caused by the flow rate, as the last two runs of the bagel structures had comparable flow rates and conversions (Figure 4). SNFs with the SiOH surface outperformed their nano-bagel counterparts at comparable flow rates (SNF SiOH entry 1 and 2, 72% conversion 0.52 µL/s; bagel SiOH entries 1–3, 68% conversion 0.56 µL/s).

### 3.3. Impact of the Catalysis Reaction on the Surface Layer

To clearly attribute catalytic activity to a specific surface morphology, it is necessary to monitor any changes on the catalytic surface caused by the process. Therefore, the nanostructured slides were cut into pieces and the surface inside the channel was compared to the outside surface by SEM to obtain the best comparison possible.

**Figure 5:**
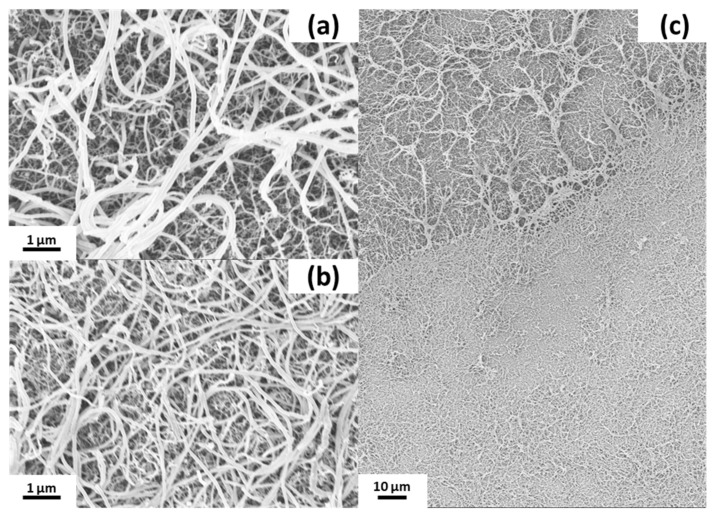
Structure of the chip after catalysis. Outside the channel (**a**), inside the channel (**b**), border area of a used Pt device (**c**), inside (**bottom**), outside (**top**).

While the SNFs clearly remained present, the inside “fluffy” structure partially collapsed (Figure 5). However, the implications of this finding are hard to assess. It is not clear if the SNFs layer structure collapsed in the liquid phase. In case it did, the question remains open if this would substantially hinder mass transfer and, finally, catalytic efficiency.

**Figure 6:**
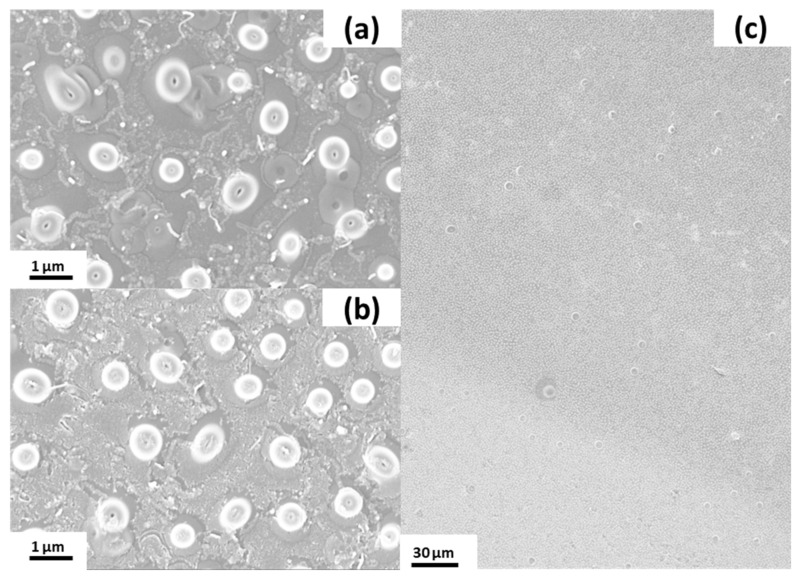
Silicone bagel structures after catalysis reaction. Outside area (**a**), channel area (**b**), comparison of outside (**top left**) to inside (**left bottom**) (**c**).

The topography and shape of the bagel structures appeared to be unaffected by the reaction conditions (Figure 6). Upon close inspection, it was observed that the coating partially broke at the edges of the bagel structures. Either this is the place most susceptible to basic hydrolysis, or the pressure excreted by the hydrogen gas formation onto this corner area is sufficiently high to introduce cracks in the coating.

### 3.4. TEM Studies of the SNF Platinum Composite Catalyst

**Figure 7:**
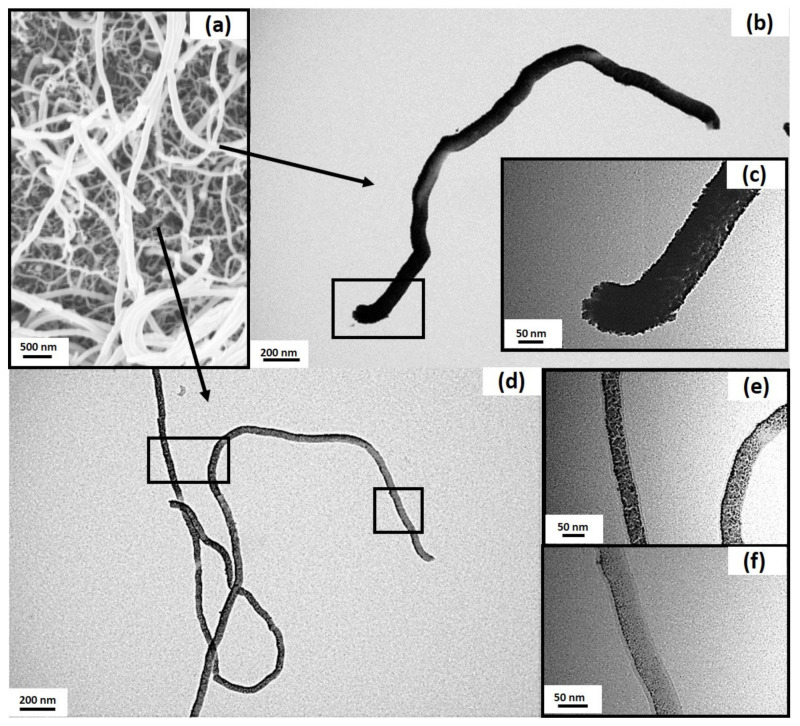
SEM of the coated surface to match the detached SNFs observed with TEM images (**a**). Large SNF (**b**) with magnified image (**c**) and smaller SNFs (**d**) with closer images at a more coated area (**e**) and a less coated area presumably heavily covered by other SNFs (**f**).

In order to display the presence and distribution of the metal catalyst on the SNFs, TEM was performed (Figure 7). The surface of all channels was sputtered with 20 nm of platinum. This thickness was measured with a quartz crystal and applied to a flat surface. SNFs, however, form, under the growth conditions applied, a surface layer in the micrometer range that amplifies the underlying surface approximately 13-fold [12]. Therefore, TEM microscopy was conducted to determine the shape in which the catalyst would be deposited.

The catalyst was clearly present in the form of nanoparticles of different sizes depending on the SNFs position in the original SNF layer (Figure 7). The larger SNFs in the SEM (a) were on average 135 ± 13 nm in diameter and reached to the top of the layer. The TEM of a large SNF (b) with 97 ± 1 nm diameter was representative of this population and was consistently heavily coated. The platinum particles at one end were 13 ± 5 nm in diameter (c). The very large particles on the tip with 18 ± 3 nm might have aggregated if the SNF had broken apart at this location during the detachment from the surface. The smaller SNFs on the bottom of the layer were found to be 55 ± 3 nm. In the TEM image, smaller SNFs with 39 ± 1 nm ((e) left SNF), 47 ± 1 nm ((e) right) and 46 ± 1 nm (f) diameter were also observed. The slight mismatch in SNF diameter between SEM and TEM might be caused by the different lighting between SEM and TEM images. The particle size decreased from the middle of the SNF with 7 ± 2 nm (e) to 4 ± 1 nm (f) at the end of the SNF.

### 3.5. Batch PNP Reduction Experiments

**Figure 8:**
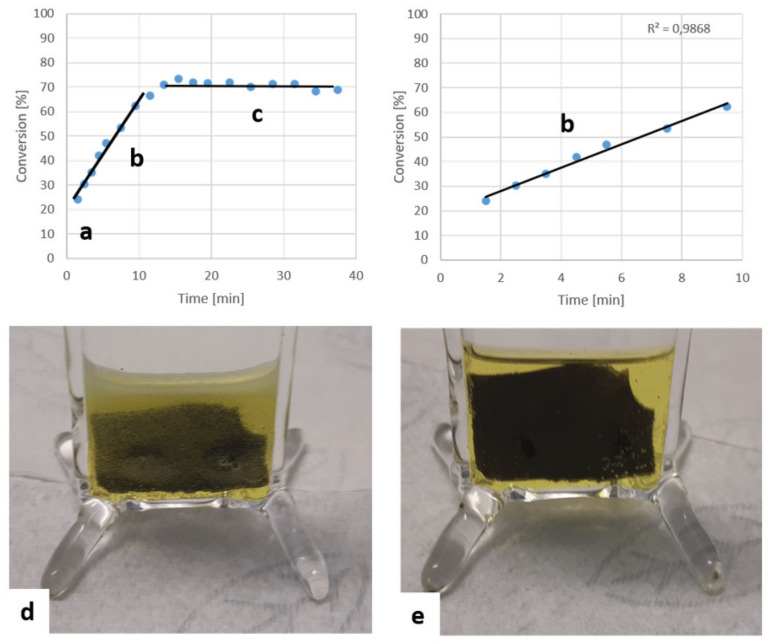
SNF-coated slide sputter coated with 20 nm of platinum. The absorption of PNP decreases as it is converted to PAP. The reaction is very fast during the first minute (**a**). Rapid hydrogen evolution is observed for about the first 6 min when using platinum (**d**). Then, the observed hydrogen evolution reduces (**e**) in the linear range of the conversion to time plot (**b**). After 12 min, the reaction rate decreases and, ultimately, the reaction stops around 15.5 min (**c**).

A batch reactor (80 × 30 × 3 mm, Figure 8d,e) was used to monitor the reaction over time. This serves as a performance comparison experiment between the flow device and traditional batch mode. Further, it would provide information on the role of the flow rate on the overall catalysis. A piece of appropriately modified glass (surface structure and metal catalyst) with surface area equal to the catalytic area of the chip (9.0 cm^2^) was submerged in a 1.7 mL 2.65 mM PNP solution containing 50 mM of NaBH_4_. Nine-microliter aliquots were periodically taken and analyzed to monitor the conversion over time.

Depending on the flow rate, it would take the open flow device between 37.5 min (0.75 µL/s) and 112 min (0.3 µL/s) to run through the entire 1.7 mL of the batch reaction. At that point, the reaction had entered the flat part of the conversion curve (c), making the total conversion rather independent of the flow rate for this reaction, in the case of platinum. Despite the excess of reducing agent (50 mM of NaBH_4_ carrying 4 hydrides to 2.65 mM of PNP requiring a 3-hydride reduction, even if only the first hydride was strong enough to perform the reduction; as the reduction potential decreases with every hydride spent, this would still amount to at least a 6.2-fold excess), the reaction did not complete. As can be seen in Figure 8d, a strong gas evolution was observed due to decay of the reducing agent in the presence of platinum.

This experiment was conducted on all the surface types previously used in open-channel devices.

**Figure 9:**
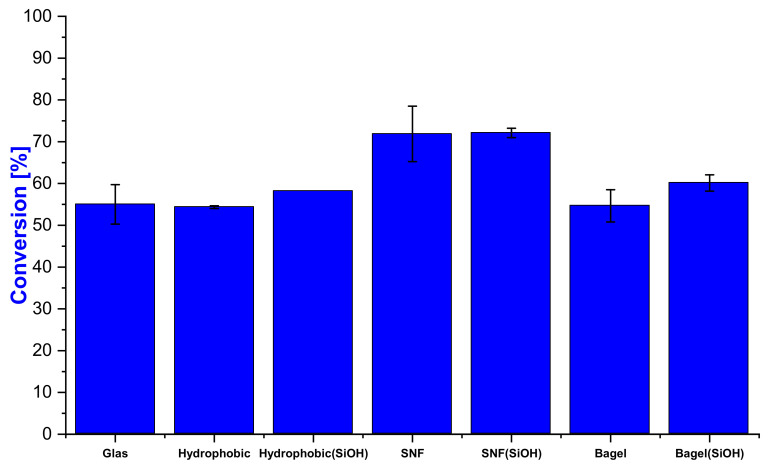
PNP conversions of the prepared surfaces in the batch mode sputtered with 20 nm of Pt catalyst.

The obtained results were in good agreement with the results obtained on free-flow devices (see Figure 4). The control surface glass (54 ± 5%), hydrophobic (54 ± 1%) and hydrophobic (SiOH) (58%) are within a few percent of each other; therefore, the surface below the 20 nm platinum layer seems not to have a large influence (Figure 9). Both SNF-based catalysts show a higher conversion than the unstructured surfaces, confirming the results of the open-flow device (Figure 4). A collapsed structure was observed under SEM identical to the channel areas of the open-flow device (see Supplementary Materials, Figure S1). Bagels had the same or slightly higher conversions than the flat control surfaces, as it was expected, due to their slightly increased surface area.

### 3.6. Metal Catalyst Screening

Using platinum as a catalyst, the reaction would not go to completion because of the competing hydrogen evolution (Figure 8). Increasing the concentration of the reducing agent would slightly improve the conversion (see Supplementary Materials, Table S1), but it would not be very efficient, considering that at least 6.2-fold excess was already used. Therefore, various metals were tested for their catalytic activity and compared to a reference to find a more active metal and to demonstrate that the advantage of SNFs is independent of the chosen catalyst.

**Figure 10:**
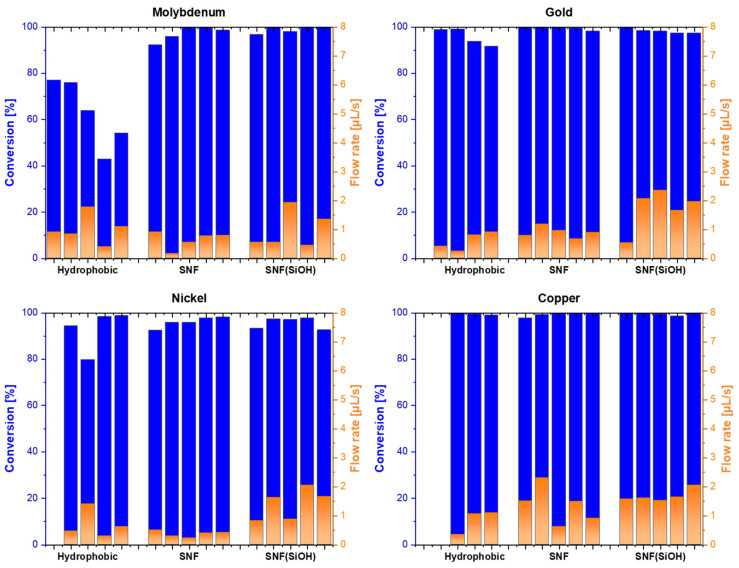
Performance of molybdenum, gold, nickel and copper catalysts (20 nm) on the open-flow device.

Molybdenum is an interesting case, since the conversion of the hydrophobic reference differed massively from either type of SNF open-channel device (Figure 10). Even higher conversions rates at higher flow rates could be achieved. Nickel showed conversions of around 95%. While the hydrophobic nickel surface had some variance in conversion, based on the flow rate, the SNF-based system had high conversion even at high flow rates. Gold exhibited even higher conversions on the hydrophobic device and essentially full conversion with SNFs and SNF(SiOH) devices at typical flow rates (0.7–1.2 µL/s) and around 95% conversion at very high flow rates (1.6–2.3 µL/s). Copper showed full conversion, no matter the surface area, and is therefore the best metal for the reaction. For the less efficient catalysts, such as platinum or molybdenum and, to some degree, nickel and gold, an increase in catalytic efficiency due to the nanostructure could be observed. To better distinguish the performances, the different metals were tested in batch mode as well. The time required for 90% of the starting material to be converted was determined to compare the fast catalyst system.

All measurements had the expected increase in catalysis provided by the increased surface area of SNFs, as reflected by the shorter time required to reach 90% conversion (Table 1). Molybdenum and gold showed very comparable conversions on glass as well as on SNFs, with ~15 min required for 90% conversion on SNFs. Nickel reached 90% conversion in 14.7 or 6.6 min, respectively. The best performing catalyst was copper, as expected based on the 100% conversion on the hydrophobic chip, which is consistent with other findings [14,30].

## 4. Summary and Outlook

In this study, the construction of an SNF- or silicone-bagel-based open-channel system for the reduction of PNP was showcased. The advantage of the additional catalytic surface inside the channels, provided by increasing the nano-roughness from none to bagels to SNFs, was demonstrated. SEM confirmed that either nanostructure was stable over five cycles and 1.1 mL of throughput. TEM microscopy of the detached SNFs showed that the catalyst is present as nanoparticles. The study was expanded to four other metals: molybdenum, gold, nickel and copper. Studies on the open-channel system gave ≥98% conversion for most catalysts, displaying the potency of the device. However, this made it difficult to evaluate the performance of the more active catalysts. Therefore, the reaction rate was monitored in a batch setup as well. The advantage of SNFs was unanimously observed with every metal tested, confirming that the advantage provided by SNFs is not limited to platinum. While most publications focus on gold, the high activity of copper is well documented [14] and molybdenum and nickel are underrepresented, considering their performance in our screening. The procedure of creating nanoparticles by sputtering could be readily expanded to the direct deposition of mixed nanoparticles [31]. High activities in the reduction of similar substrates with mixed metal particles have been reported [32]. This could occur either from an alloy target or by sputtering from two different targets simultaneously. Mixed-metal particles of zinc or aluminum with a catalytic metal could be sputtered and further activated to create Urushibara-type [33] or Raney-type [34] catalysts. Further, the SNF could be infused with a liquid to improve the catalyst deposition throughout the SNF layer, similar to the technique used to harvest nanoparticles from the sputtering process [31,35].

## Figures and Tables

**Table 1:** Total conversion and time for 90% conversion in batch mode with 20 nm of catalyst.

Surface Catalyst	Glass Molybdenum	SNF(SiOH) Molybdenum	Glass Platinum	SNF(SiOH) Platinum	Glass Nickel	SNF(SiOH)Nickel	Glass Copper	SNF(SiOH) Copper	Glass Gold	SNF(SiOH) Gold
Final Conversion [T = 37.5 min]	82.3 ± 9.5%	99.6 ± 0.2%	55.0 ± 4.7%	72.1 ± 1.1%	98.9 ± 0.4%	98.9 ± 0.8%	99.8 ± 0.2%	99.7 ± 0.3%	97 ± 1%	99.0 ± 0.3%
Time required for 90% conversion [min]	27.5	14.7 ± 0.3	-	-	14.7 ± 2.3	6.6 ± 1.2	5.6 ± 0.8	1.2 ± 0.2	17.6 ± 0.3	14.9 ± 1.2

## Data Availability

Any further data can be provided upon personal request.

## Supplementary Materials

The following are available online at https://www.mdpi.com/article/10.3390/nano11071663/s1, Equation (S1): Calculation of the catalytic surface to reagent ratio, Figure S1: SNFs before and after catalysis in the batch system, Table S1: Impact of the NaBH_4_ concentration on conversion and flow rate.
