# Supplementary material for: Silicone Nanofilament Support Layers in an Open-Channel System for the Fast Reduction of Para-Nitrophenol
Source: Nanomaterials (Basel). 2021 Jun 24;11(7):1663. doi: 10.3390/nano11071663 (PMC8305141; doi:10.3390/nano11071663)
Supplement: Supplementary file 1 [file nanomaterials-11-01663-s001.zip › nanomaterials-1244137-supplementary.pdf]

Supporting information

Silicone Nanofilament Support Layers in an Open-Channel System for the Fast Reduction of *Para*-Nitrophenol

Noah U. Naef and Stefan Seeger *

Department of Chemistry, University of Zurich, 8057 Zurich, Switzerland; noah.naef@chem.uzh.ch
* Correspondence: sseeger@chem.uzh.ch

Equation S1 calculation of the catalytic surface to reagent ratio:

$$\frac{Catalyst\ surface\ (9\ cm^2)}{System\ volume\ (73\ \mu L)\ \cdot\ PNP\ concentration\ (2.65\ mM)} = 46.5\ \frac{cm^2}{\mu mol\ PNP} \tag{S1}$$

Different $NaBH_4$ concentrations were tested.

Table S1. Impact of the $NaBH_4$ concentration on conversion and flow rate.

Sodium borohydrid concentration	Conversion	Flow rate
100 mM	82 %	0.98 µL/s
50 mM	77 %	0.75 µL/s
25 mM	52 %	0.43 µL/s

The gas formation instigated by the reducing agent heavily affected the flow rate, as previously described. The increase from 25 mM to 50 mM (general condition used for all experiments unless stated otherwise) increased the conversion by 25% (see Table 2). The reaction at 100 mM $NaBH_4$ had an exceedingly violent hydrogen evolution and increased the conversion by only 5%. While the $NaBH_4$ concentration clearly influences the conversion, the 50 mM concentration is close to the ideal condition.

SNFs before and after catalysis in the batch system

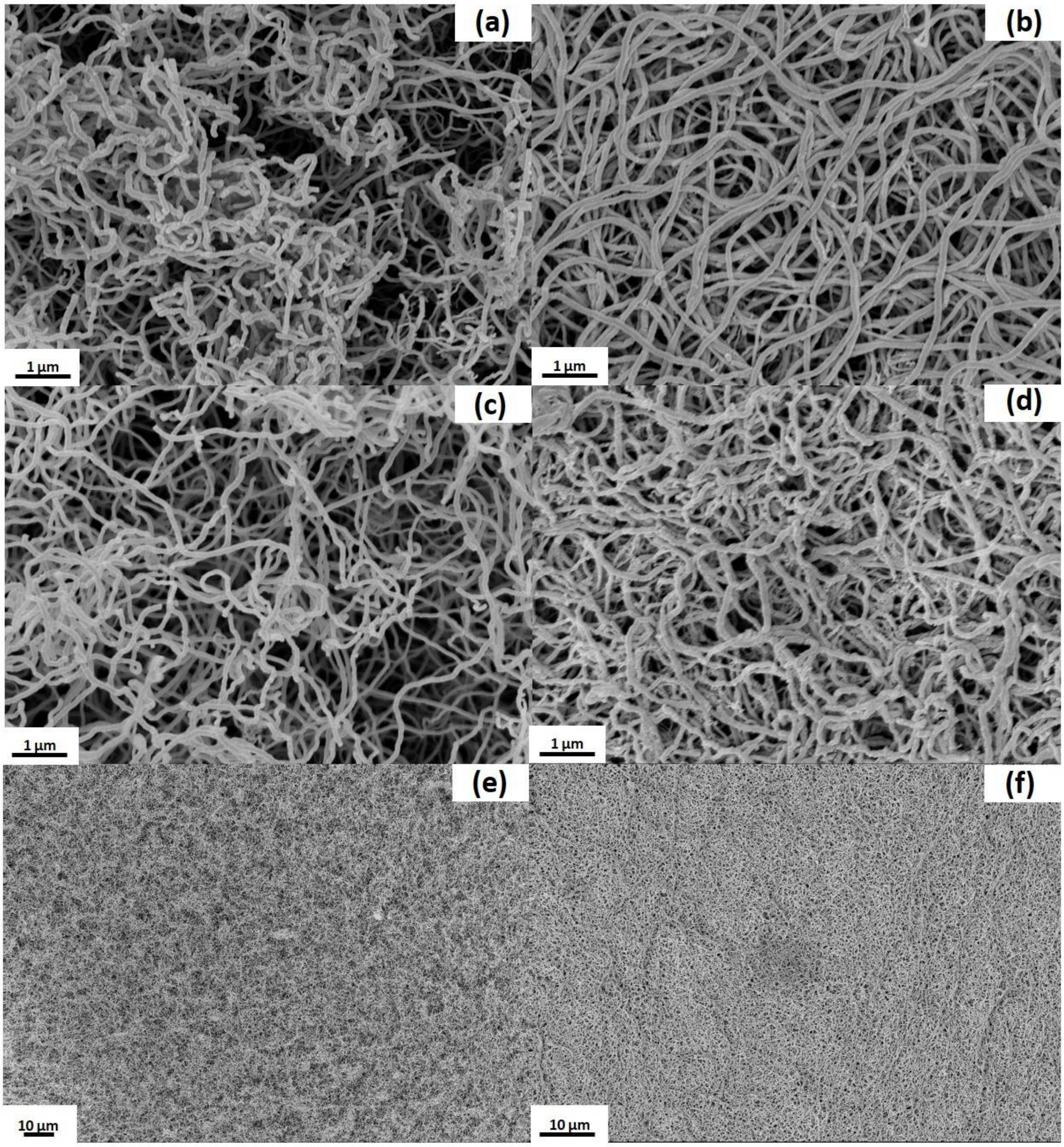

Figure S1. SEM of SNF sputtered with 20 nm Pt before catalysis in batch (a) after (b). SNF(SiOH) with 20 nm Pt before catalysis in batch (c) after (d). Large magnification SNF(SiOH) before (e) and after (f) catalysis.
